# The long-term prognosis of epilepsy patients with medically treated over a period of eight years in Turkey

**DOI:** 10.12669/pjms.334.13194

**Published:** 2017

**Authors:** Pelin Duman, Asuman Orhan Varoglu, Esra Kurum

**Affiliations:** 1 Pelin Duman, Department of Neurology, Medical School, Medeniyet University, Istanbul, Turkey; 2 Asuman Orhan Varoglu, Department of Neurology, Medical School, Medeniyet University, Istanbul, Turkey; 3 Esra Kurum, Department of Statistics, University of California, Riverside -USA

**Keywords:** Prognosis, Epilepsy, Antiepileptic drug (AED), Seizure frequency, Clinical characteristics

## Abstract

**Objective:**

The aim of this study was to investigate the effect of demographic and clinical characteristics on temporal changes in seizure control and frequency in medically treated epilepsy patients to guide treatment modalities.

**Methods:**

We retrospectively analyzed the association between clinical and demographic characteristics and seizure frequency in 1329 epilepsy patients who were followed up at an outpatient clinic for one to eight years, 2008-2015..

**Results:**

Younger age at first seizure (*p* = 0.0465) and a long disease duration (*p* = 0.0406) had a negative effect on seizure control in all the epilepsy patients. Febrile convulsions (FCs) (*p* > 0.0001), perinatal risk (*P*NR) (*p* > 0.0002), a family history of epilepsy (FHE) (*p* > 0.0016), antiepileptic drug (AED) use (*p* > 0.001), mental retardation (MR) (*p* > 0.001), and psychiatric disorders (*p* > 0.0478) were prognostic indictors of temporal changes in seizure frequency. The presence of PNR (*p* = 0.0416), age at onset of epilepsy (*p* = 0.034), central nervous system infection (CNSI) (*p* = 0.04), and AEDs number (*p* = 0.0282) were prognostic indicators of not remaining seizure free for one year. In those with partial epilepsy, a trauma history (*p* = 0.05), a longer epilepsy duration (*p* = 0.0057), and FHE (*p* = 0.0466) increased the frequency of seizures, whereas cerebrovascular event (CVE) history decreased the seizure frequency (*p* = 0.0413). In addition, FHE (*p* = 0.0438) and psychotic disorders (*p* = 0.0416) increased generalized seizures frequency.

**Conclusion:**

In all the epilepsy patients, a younger age at onset and longer duration of epilepsy were associated with a poor prognosis. The presence of PNR, age at onset of epilepsy, CNSI, and AEDs numbers were prognostic indicators of not remaining seizure free for one year. Increasing AEDs number was not effective in controlling seizures in partial epilepsy, but it was effective in controlling seizures in generalized epilepsy.

## INTRODUCTION

Epilepsy is a common neurological disorder worldwide.^1^ Information on the patient’s prognosis is necessary to prevent unnecessary drug use. There are only a few reports on the prognostic factors in patients with medically treated epilepsy.^2^,^3^ However, the number of patients, evaluated risk factors, and follow-up of the patients were very limited in these reports. No published studies have investigated temporal changes in factors that can affect the prognosis of epilepsy patients. Thus, the present study retrospectively studied the effects of demographic and clinical characteristics of epileptic patients treated medically on temporal changes in the frequency of seizures during an eight-year follow-up study.

## METHODS

We retrospectively analyzed 1329 epilepsy patients who were followed up at an outpatient clinic for one to eight years, 2008-2015. All the patients were diagnosed according to the seizure semiological criteria proposed by the International League against Epilepsy,^4^ in addition to electroencephalography (EEG) and magnetic resonance imaging (MRI) findings. Patients younger than 18 years and older than 80 were excluded from the study.

The hospital charts and database were reviewed to obtain information on the patients’ clinical histories Frequency of different types of the seizures each month was recorded (Table-I).

**Table-I:** The demographic and clinical characteristics in all epilepsy patients.

		*No.*	*%*
Gender	Male	649	48,8
	Female	679	51,2
Handedness	Right	645	88,4
	Left	7	10,4
	Mixed	9	1,2
Mental Retardion	Absent	776	70,8
	Mild	185	16,9
	Moderate	83	7,6
	Severe	52	4,7
Perinatal Injury	Absent	1172	89,1
	Present	143	10,9
	Absent	1023	77,9
Family history of epilepsy	Present	291	22,1
	Absent	1099	83,6
Head Trauma	Present	215	16,4
	Absent	1224	93,2
Central nervous system infection	Present	90	6,8
	Absent	1089	82,9
Febrile convulsion	Present	225	17,1
	Absent	1126	96,2
Consanguinity between parents	Present	181	3,8
	Absent	1238	94,4
History of status epilepticus	Present	74	5,7
	Normal	759	60
Cranial MRI	Unilateral sclerosis	90	7,1
	Bileteral sclerosis	5	0,4
	Encephalomasia	307	24,3
	Vascular pathology	19	1,5
	Others	85	6,7
EEG	Normal	494	12,3
	Unidentified	31	2,5
	Epileptiform or paroksismal activity on the pathological side	249	19,4
	Epileptiform or paroksismal activity on the nonpatholojical side	46	3,6
	Generalized epileptiform or paroksismal activity	452	35,3
SPS	Absent	1142	86,4
	Present	179	13,6
CPS	Absent	888	43,9
	Present	433	32,8
Aura	Absent	929	71,2
	Present	376	28,8
Primary GTCS	Present	740	44
Secondary GTCS	Present	362	27,3

SPS: Simple partial seizures; CPS: Complex partial seizures; GTCS: Generalized tonic clonic seizures.

Models were developed to determine the effects of demographic and clinical characteristics on the frequency of SPSs, CPSs, aura , and primary and secondary GTCSs seizures in all epilepsy patients.

In the models, the following factors were analyzed: The effects of demographic and clinical characteristics on temporal changes in all the epilepsy frequency and in the focal (SPS, CPS, and aura) epilepsy frequency and in the generalized epilepsy (primary and secondary GTCSs) frequency and in patients with at least one seizure experience per year seizure frequency (annual seizure average >0.08 per month, in other words, patient with not remaining seizure free for one year).

### Statistical Analysis

The Statistical Package for Social Sciences for Windows vs. 16.0 program was used for the statistical analysis. Descriptive statistical methods were used. A logistic regression analysis was used to analyze categorical data. Quantitative data were analyzed by ordinal regression analysis.

Models were created to avoid multiple correlations between independent variables. The Akaike information criteria (AIC) scale was used to determine which variables to include in the models. The relationship between the categorical variables and time was determined by comparing the mean numbers of seizures in the first, second, third, fourth, fifth, sixth, seventh, and eighth years. Data are presented as mean ± standard deviation. A *p*-value of <0.05 was considered significant.

## RESULTS

The study consisted of 679 females and 649 males aged 18 to 80 years. Four hundred fifty-seven patients were single, 496 patients were married, and 32 patients were divorced. One hundred eighty-six patients had little education. Seven hundred thirty-four patients had graduated from primary and high schools, and 74 patients had graduated from universities. The subtypes of epilepsy were as follows: idiopathic (*n* = 349, 26.7%); cryptogenic (*n* = 364, 27.8%), and symptomatic (*n* = 595, 45.5%). Psychiatric disorders were present in 338 (26%) patients. Systemic disturbances history was noted in 337 (25.5%) patients. One hundred-thirty (9.9%) patients had intracranial surgery history, and 114 (8.7%) had CVE history. Seventy-four (5.7%) patients experienced status epilepticus. Only one type of seizure was present in 940 (71.2%) patients, two types of seizures were present in 369 (27.9%) patients, and three types of seizures were present in 12 (0.9%) patients.

Three AIC models were developed to analyze the effects of demographic and clinical characteristics on temporal changes in the total seizure frequency in all the epilepsy patients (Table-II).

**Table-II:** The effects of demographic and clinical characteristics on temporal changes in the total seizure frequency in all the epilepsy patients.

	*I. Follow-up mean ± SD*	*II. Follow-up mean ± SD*	*III. Follow-up mean ± SD*	*IV. Follow-up mean ± SD*	*V. Follow-up mean ± SD*	*VI. Follow-up mean ± SD*	*VII. Follow-up mean ± SD*	*p value*
FC	14.104	49.029	58.675	40.796	46.547	-14.206	-0.559	<0.0001
	±1.1152	±1.5729	±1.9718	±2.3383	± 2.7579	± 3.7249	± 4.4674	
PNR	1.447	10.714	-0.5849	0.7283	-0.3518	167.632		0.0002
	±1.4674	±2.2706	± 2.8406	± 3.3128	± 3.8634	± 5.0393		
FHE	-0.939	-5.096	-44.383	-52.388	-3.028	-15.759	17.812	0.0016
	± 1.2633	±1.8210	±2.2847	±2.6850	±3.6052	±5.0506	±6.2024	
AED	0.1586	-20.494	-11.722	0.0122	0.8456	0.9376	-0.7565	0.001
	±0.5151	±0.7246	±0.8907	±0.9854	±1.1590	±1.5002	±14427	
MR(Mild)	-0.374	0.0006	0.1473	0.5954	0.0570		0.0761	<0.001
	0.2381	0.3463	0.4392	0.5311	0.6249	0.7749	0.9045	
Moderate	-0.170	-0.082	0.1473	0.5954	0.0570	-0.5945	-1.159	<0.001
	0.3272	0.4780	0.4392	0.5311	0.624	0.7749	11.764	
Severe	-0.565	-1.956	-36.045	-42.118	35.638	-32.675	-13.729	<0.001
	0.3933	0.5774	0.7342	0.8981	10.764	12.992	15.529	
PD	-1.933	-3.836	-43.883	0.2829	19.505	41.319	0.0478	0.0478
	17.692	24.428	32.382	39.628	46.097	95.414		

FC: febril convulsion; PNR: perinatal risk; FHE: family history of epilepsy; AED: antiepileptic drugs; MR: mental retardation; PD: psychiatric disorders.

***Model I*** analyzed the effects of gender, marital status; epilepsy duration; FHE; PNR history; CNSI history, FCs, aura, and psychiatric disorders; consanguinity between parents; and AEDs numbers.

***Model II*** analyzed the effects of MR, CVE, and trauma.

***Model III*** analyzed the effects of abnormal EEG findings, hand dominance, and systematic disease history.

The age of onset of epilepsy (0.3194 ± 0.1559 years) (*p* = 0.0465) and epilepsy duration (0.03323 ± 0.01620) (*p* = 0.0406) affected the prognosis of all the epilepsy patients. The seizure frequency increased in association with aging in patients with depression and in the first years of follow-up in patients with psychotic disorders, personality disorders, and other psychiatric disorders (*p* = 0.047).

Three AIC modes were also developed to analyze the effects of demographic and clinical characteristics on temporal changes in SPS, CPS, and aura frequency (Table-III).

**Table-III:** The effects of demographic and clinical characteristics on temporal changes in focal (SPS, CPS, and aura) epilepsy frequency.

*Clinical characteristic feature*	*Mean*	*Odd ratio*	*p value*
Family history of epilepsy	-0.4287	0.6514	0.0466
CVE	-1.1563	0.3146	0.0413
Duration of epilepsy	0.0334	1.0340	0.0057
Trauma	0.4408	1.5540	0.050

CVE: cerebrovascular event.

***Model I*** analyzed the effects of the duration of epilepsy, consanguinity between parents, CNSI, FHE , PNR, psychiatric disorders, FCs, and AED use on seizure frequency.

***Model II*** analyzed the association of MR, CVE history, trauma, and epilepsy duration with seizure frequency, as well as the effect of trauma and MR in seizure frequency.

***Model III*** analyzed the effects of abnormal EEG findings and systemic disease history on seizure frequency.

AED number showed a significant association with SPS, CPS and aura (odds ratio [OR]: 1.779, *p* < 0.0001).

Two AIC models were developed to analyze the effects of demographic and clinical characteristics on temporal changes in the frequency of primary and secondary GTCSs (Table-IV).

**Table-IV:** The effects of demographic and clinical characteristics in temporal changes in the frequency of generalized (primary and secondary GTCSs) epilepsy.

*Clinical characteristic feature*	*mean*	*Odd ratio*	*p value*
Family history of epilepsy	0.6135	1.8469	0.0438
Psychiatric disorder-depression	-0.7202	0.4867	0.0416
Psychiatric disorder-psychosis	0.4517	1.5710	

***Model I*** analyzed the association of gender, consanguinity between parents, epilepsy duration, age at first seizure onset, FHE, PNR, CNSI, marital status, abnormal EEG findings, psychiatric disorders, and AED use with seizure frequency. This model also analyzed the association of CNSI, PNR, FHE, consanguinity between parents, and abnormal EEG findings with changes in seizure frequency.

***Model II*** analyzed the association of FCs, MR, CVE, trauma and systemic disease history with seizure frequency.

Primary and secondary GTCSs decreased (51%) in patients with depression (OR: 0.4867) and increased (57%) in patients with psychosis (OR: 1.5710, *p* = 0.0416).

Two AIC models were developed to analyze the effects of demographic and clinical characteristics on patients with at least one seizure experience per year (annual seizure average > 0.08 per month). (Table-V).

**Table-V:** The effects of demographic and clinical characteristics of patients with not remaining seizure free for one year.

*Clinical characteristic feature*	*Mean*	*Odd ratio*	*p value*
Age of onset epilepsy	-0.0172	0.9829	0.0344
PNR	0.4110	1.5083	0.0416
CNSI	0.6703	1.9548	0.0400
AED	1.0731	2.9244	0.0282

PNR: perinatal risk, AED: antiepileptic drug, CNSI: central nervous system infection.

***Model I*** analyzed the associations between gender, marital status, epilepsy duration, age of onset of epilepsy, FHE, PNR, CNSI, FCs, consanguinity between parents, psychiatric disorders, and AED use.

In addition, the effects of epilepsy duration, FHE, PNR, CNSI, FCs, consanguinity between parents, and psychiatric disorders were analyzed in patients who not remained seizure free for one year.

***Model II*** analyzed the association of abnormal EEG findings, trauma, and systemic disease history.

## DISCUSSION

In our study, the age of seizure onset and epilepsy duration affected the prognosis in all the epilepsy patients. We also found that a younger age at onset of seizures was associated with a reduced chance of remaining seizure free for one year. Similar to our findings, Kim et al.^5^ reported that a younger age at the onset of seizures was associated with a poor prognosis. However, other reports found that the onset of seizures at a young age was associated with a good prognosis.^5^,^6^ The findings of our study, which showed that a longer duration of epilepsy was associated with a poorer prognosis, are consistent with those of Sperk et al.^7^ Thus, a young age at seizure onset and longer epilepsy duration appear to be consistently always associated with a poor prognosis in all epilepsy, despite changes in other parameters over time. In the present study, in all the epilepsy patients, FHE, PNR, FCs, psychiatric disorders, MR, and AED use affected temporal changes in seizure frequency.

We have observed that in the presence of FHE, the seizure frequency increased over time in cases of generalized epilepsy and decreased in cases of complex partial epilepsy. Another study stated that FHE was not a prognostic factor in mesial temporal epilepsy.^1^ Supporting our findings a previous study reported that genetic mechanisms in inherited and sporadic epilepsies can increase drug seizure frequency by causing drug resistance.^8^

We found that the epilepsy duration increased the partial seizures frequency but not generalized seizures frequency. The loss of volume in the hippocampus was shown to be correlated with the disease duration, with progressive hippocampus and loss of neurons causing volume loss in the temporal lobe, resulting in recurrent seizures.^9^ Similar to our findings, previous research found no significant association between prognosis and generalized epilepsy frequency.^10^ Gomez-Ibañez et al.^11^ showed that long epilepsy duration was associated with a poor prognosis in refractory idiopathic generalized epilepsy. The results suggest that the prognosis of each epilepsy syndrome is related to the characteristics of that syndrome.

In our study, in generalized epilepsy patients, the presence of psychosis increased the seizure frequency over time. This may have been the result of an interaction between the genetic mechanisms responsible for idiopathic epilepsy and the antipsychotic drugs used. In all the epilepsy patients, the presence of depression was associated with a poor prognosis in advancing years. In the literature, both idiopathic epilepsy^11^ and complex partial seizures^12^ were reported to be associated with a poor prognosis.

We found a significant relationship between the presence of trauma and partial seizures frequency. Annegers et al.^13^ reported that the risk of developing epilepsy was greater in patients who experienced major head trauma than in those with mild to moderate head trauma. Another study showed that seizure control in patients with epilepsy secondary to traumatic brain injury was worse than in patients without such injury.^14^

According to our analysis, CVE history reduced partial seizures frequency by 69%. This suggests that some partial seizures caused by CVE and treated by AEDs in the acute phase may arise due to hemodynamic changes secondary to CVE and metabolic imbalance. Mohanraj and Brodie^14^ showed that 70% of patients who developed epilepsy due to CVE achieved full seizure control.

We found no significant correlation between abnormal cranial imaging findings and partial and generalized seizures frequency. Previous studies have showed that abnormal cranial imaging findings were associated with increased seizures frequency.^14^,^15^ In our study, the presence of FC was associated with temporal changes in seizure frequency in all epilepsy patients. A previous study of surgical and nonsurgical treatment found that FC had no effect on seizure frequency.^1^ In contrast to this report, another study found that the presence of FCs was associated with a poor prognosis.^5^

In the present study, the presence of PNR seemed to have an adverse effect on seizure control in later years but not in the years shortly after the diagnosis of epilepsy. The presence of PNR also seemed to be associated with temporal changes in seizure frequency. Furthermore, the presence of PNR reduced the chance of remaining seizure free for one year. Kim et al.^16^ reported that older age at onset of nonlesional temporal epilepsy was associated with a better prognosis, similar to our findings. Some studies conducted in developed countries finding no relation between PNR and the prognosis in epilepsy patients and others conducted in developing countries finding that PNR was a poor prognostic indicator.^14^,^15^

According to our study, severe MR was not a prognostic indicator. Seizure control is difficult with advancing years in patients with mild to moderate mental retardation. Such difficulty may be related to treatment compliance. Sillanpaa et al.^3^ observed a significant association between the presence of MR and poor seizure control. Contrary to this published report, Kumlien et al.^6^ reported that IQ had no effect on prognosis.

We found that patients with CNSI history had a low chance of remaining seizure free for one year. However, the prognosis was not bad in all epilepsy patients in the presence of CNSI. Ohtsuka et al.^17^ suggested that encephalitis may be involved in the development of multifocal epileptic foci in more than one region of the brain in the acute phase of epilepsy, potentially leading to uncontrolled seizures.

In the present study, we found a 77% increase in the frequency of partial seizures in accordance with each increase in the number of drugs. We found that seizure control is not parallel to the increase in AED count in partial seizures. However, we did not observe this effect in cases of idiopathic generalized epilepsy. Kwan reported that 525 patients seizure control provided by first, second, and third treatment regimens was 47%, 13%, and 1%, respectively.^18^

## CONCLUSION

In the present study, younger age at epilepsy onset and a longer duration of epilepsy were associated with a worse prognosis in all the epilepsy patients. In complex partial epilepsy, FHE, duration of epilepsy, trauma, and CVE resulted in temporal changes in seizure control. In addition, the AED count was related to poor seizure outcomes in complex partial epilepsy patients. In generalized epilepsy, FHE and psychiatric disorders led to temporal changes in seizure control. In all patients with epilepsy, we identified the presence of PNR, age at onset of epilepsy, CNSI, and the AED count as prognostic indicators of not remaining seizure free for one year.

### Author’s Contribution

Pelin Duman did data collection and manuscript writing.

Asuman Orhan Varoglu did manuscript writing and takes the responsibility and is accountable for all aspects of the work in ensuring that questions related to the accuracy or integrity of any part of the work are appropriately investigated and resolved.

Esra Kurum designed and did statistical analysis.
